# Mimicking Metabolic Disturbance in Establishing Animal Models of Heart Failure With Preserved Ejection Fraction

**DOI:** 10.3389/fphys.2022.879214

**Published:** 2022-05-03

**Authors:** Hui Li, Yi-Yuan Xia, Chun-Lei Xia, Zheng Li, Yi Shi, Xiao-Bo Li, Jun-Xia Zhang

**Affiliations:** ^1^ Department of Cardiology, Nanjing First Hospital, Nanjing Medical University, Nanjing, China; ^2^ Department of Intensive Medicine, The Affiliated Jiangning Hospital of Nanjing Medical University, Nanjing, China

**Keywords:** heart failure with preserved ejection fraction, metabolic disturbance, animal models, ventricular diastolic dysfunction, clinical translation

## Abstract

Heart failure (HF), the terminal state of different heart diseases, imposed a significant health care burden worldwide. It is the last battlefield in dealing with cardiovascular diseases. HF with preserved ejection fraction (HFpEF) is a type of HF in which the symptoms and signs of HF are mainly ascribed to diastolic dysfunction of left ventricle, whereas systolic function is normal or near-normal. Compared to HF with reduced ejection fraction (HFrEF), the diagnosis and treatment of HFpEF have made limited progress, partly due to the lack of suitable animal models for translational studies in the past. Given metabolic disturbance and inflammatory burden contribute to HFpEF pathogenesis, recent years have witnessed emerging studies focusing on construction of animal models with HFpEF phenotype by mimicking metabolic disorders. These models prefer to recapitulate the metabolic disorders and endothelial dysfunction, leading to the more detailed understanding of the entity. In this review, we summarize the currently available animal models of HFpEF with metabolic disorders, as well as their advantages and disadvantages as tools for translational studies.

## Introduction

Heart failure (HF) refers to a complex clinical syndrome when cardiac output failed to meet the body’s need. The typical symptoms and signs include dyspnea, fatigue, lower limb edema and so on (Witte and Clark, 2007). At age of 40 years, the lifetime risk for HF was nearly 20% for both men and women (Lloyd-Jones et al., 2002). Despite considerable advances in clinical management and treatment, morbidity and mortality of HF remain high after initial diagnosis (Metra and Teerlink, 2017).

The understandings of HF are dynamic and new challenges continue to emerge. Considering any cardiac structural and/or functional dysfunctions might contribute to HF, updated classification of HF is proposed according to left ventricular ejection fraction (LVEF) (Writing Committee et al., 2021). The four classes of HF are HF with reduced ejection fraction (HFrEF), where LVEF ≤40%; HF with median range ejection fraction (HFmrEF), where LVEF ranged from 41% to 49%; HF with preserved ejection fraction (HFpEF), where LVEF ≥50%; and HF with improved ejection fraction (HFimpEF), where LVEF ≤40% at baseline, but increase ≥10% from baseline and LVEF >40% at the second measurement.

Notably, HFpEF and HFrEF are the two most common types of HF. HFrEF has an overt decreasing of myocardial systolic capacity, that is often caused by myocardial infarction, hypertension, heart valvular diseases and dilated cardiomyopathy. Due to the relatively clear etiology of HFrEF, many animal models have been established for HFrEF researches (Noll et al., 2020).

The surgically induced HFrEF models are most widely used in mechanistic studies. For example, the hallmark murine model with thoracic aorta constriction (TAC) reported by Rockman (Rockman et al., 1991; Rockman et al., 1994), which became the standard surgery to construct stress-load-induced HFrEF. Post-myocardial infarction model created by the left anterior descending artery (LAD) ligation is usually adopted for exploring the mechanisms and treatment alternatives for ischemic HF (Sawall et al., 2017). Moreover, the pulmonary artery constriction-induced right ventricular hypertrophy and right HF model was successfully established to evaluate pathophysiological mechanisms of right ventricular dysfunction (Rockman et al., 1994; Wang et al., 2019). Furthermore, uninephrectomy, combined with angiotensin II and salt loading were applied in mice to recreate HF model associated with hypertensive heart disease (Tsukamoto et al., 2013).

Besides that, there are HFrEF models established by single medicine induction, including isoproterenol (Chang et al., 2018), angiotensin II (Pan G et al., 2018), alcohol (Guo and Ren, 2010) and doxorubin (Zeiss et al., 2019). These models have been adopted in study of specific intrinsic or extrinsic stimulus-induced HF. Furthermore, gene editing models were used in HFrEF model construction, given that genetic factors play an important role in development of dilated cardiomyopathy. For example, RNA-binding motif protein-20 (RBM20) gene-edited pigs (Schneider et al., 2020) and myosin binding protein C knockout mouse model (MYBPC3^−/−^) (Harris et al., 2002) showed HFrEF phenotypes. These models have facilitated the development of molecular diagnosis and targeted therapeutic approaches for HFrEF.

## Metabolic Syndrome and HFpEF

HFpEF accounts for about 50% of all cases with HF diagnosis. However, there is a scarcity of definite etiologies and unanimous clinical diagnostic criteria for HFpEF. Furthermore, eﬀective therapeutic alternatives, such as inhibitors of the renin-angiotensin system, beta blockers, aldosterone inhibitors and neprylisin inhibitors (Ferrari et al., 2020) for HFrEF, have failed to demonstrate improvements in mortality for patients with HFpEF.

An impediment for developing effective therapies comes from the diversity of clinical comorbidities and heterogeneities of HFpEF patients. However, many comorbidities of HFpEF are metabolic disorders. Metabolic syndrome is the aggregate of cardiovascular risk factors comprising central and abdominal obesity, systemic hypertension, insulin resistance (or type 2 diabetes mellitus), and atherogenic dyslipidemia. These conditions are interrelated and share underlying mediators (Eckel et al., 2005). Metabolic disorders are associated with altered substrate utilization and energy transduction in the myocardium and skeletal muscles. Energy molecule adenosine triphosphate (ATP) production is reduced due to mitochondrial free radical damage in HFpEF (Bates et al., 2014). It is suggested that increased glycolysis is the earliest energy metabolic change during HFpEF development. However, uncoupling of glycolysis and glucose oxidation may occur in HFpEF (Fillmore et al., 2018). In a metabolic risk-related rat model of HFpEF, the free mitochondrial calcium concentration is higher in HFpEF owing to alterations in mitochondrial and cytosolic Ca^2+^ handling. it might trigger mitochondrial permeability transition pore opening and reduced ATP production (Miranda-Silva et al., 2020).In addition, Ca^2+^ reuptake by sarcoplasmic reticulum in cardiomyocytes is primarily dependent on Ca^2+^ -ATPase (SERCA2a). Increased levels of reactive oxygen species (ROS) in cardiomyocytes impaired the phosphorylation of SERCA2a, and reduced the ability of the sarcoplasmic reuptake of Ca^2+^(Gómez-Viquez et al., 2021; McCandless et al., 2022), leading to intracellular Ca^2+^ overload and diastolic dysfunction (Runte et al., 2017; Silverman et al., 2020).

Insulin resistance also induces endothelial dysfunction. In obese rats with HFpEF, arginine-metabolizing enzymes were noticed to be increased in blood and heart, and nitric oxide (NO) turnover was reduced (Büttner et al., 2021). PROMIS-HFpEF study demonstrated a high prevalence of coronary microvascular dysfunction in HFpEF without unrevascularized macrovascular stenosis (Shah et al., 2018).

**FIGURE 1:**
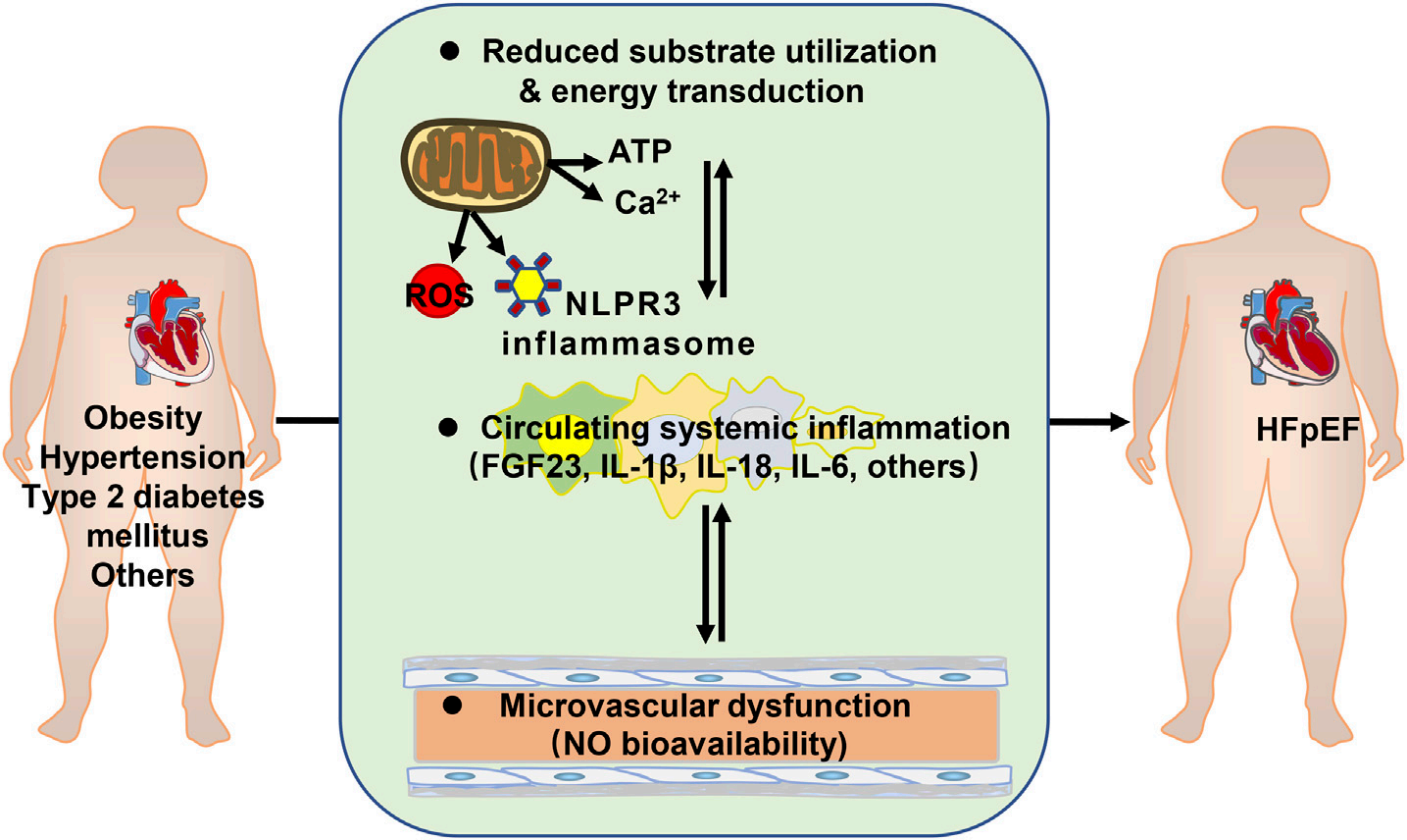
Metabolic disorders contributing to heart failure with preserved ejection fraction (HFpEF). ATP: adenosine triphosphate; ROS: reactive oxygen species; FGF23: fibroblast growth factor 23; IL: interleukin; NO: nitric oxide.

Chronic low-level metabolic inflammation (Withaar et al., 2021a; Withaar et al., 2021b; Mishra and Kass, 2021) is predisposed to the development of HFpEF as well. The mouse model of HFpEF, increased assembly of NLPR3 inflammasome on hyperacetylated mitochondria contributes to overproduction of interleukin-1β/IL-18 and tissue fibrosis (Deng et al., 2021). Plasma fibroblast growth factor 23 (FGF23) is higher in HFpEF patients and is strongly associated with exercise incapacity and prognosis (Kanagala et al., 2020). Plasma IL-6 level is associated with an increased risk of developing HFpEF in community-dwelling individuals, independent of potential confounders. (Chia et al., 2021) (Figure 1).

Machine learning and cluster analysis were applied to identify similar patients based on their combined features. In a previous study, female patients were divided into three groups. The first group had a low proportion of vascular risk factors. The second group had a higher proportion of hypertension and diabetes, but lower proportion of kidney disease and diastolic dysfunction. The third group had higher rates of atrial ﬁbrillation (AF) and chronic kidney disease and worst long-term outcomes. Similarly, three groups of male patients were identified by machine learning. The first group had a high proportion of coronary artery disease (CAD), dyslipidemia, higher serum creatinine, and diastolic dysfunction. The second group had highest BMI, and high proportion of CAD, obstructive sleep apnea, and poorly controlled diabetes. The third group had high rates of AF, elevated brain natriuretic peptide (BNP) and biventricular remodeling with a higher cardiovascular mortality (Nouraei and Rabkin, 2021). The high prevalence of metabolic disorders suggests metabolic diseases should be taken into consideration during constructing appropriate animal models for clinical translation. Herein, we reviewed animal models for HFpEF with metabolic disorders (Table 1).

**TABLE 1:** Animal models for heart failure with preserved ejection fraction and metabolic disorders.

Species	Year	First author	Methods	Age	Modeling cycle	Strain	Gene editing	Sex	Imaging and functional evaluation	Advantages	Disadvantages
Mouse	2016	María Valero-Muñoz	Uninephrectom; d-aldosterone (0.30 μg/h) × 4 weeks	8 weeks	4 weeks	C57BL/6J	NA	Male	E↑ A↑ DT↓ IVRT↓	Shortening of modeling period	Being difficult to operate
Mouse	2016	Cong Wang	STZ (45 mg/kg) × 5 days	12 weeks	3 weeks	C57BL/6	ALDH2−/−	Male	E/A↓ E/E’↑ E’↓ DT↑		Long reproductive cycle and high feeding cost
Mouse	2017	Qingqing Meng	60%HFD	6–12 weeks	16–20 weeks	AKR/J	NA	Male	RVSP↑ mPAP↑ LVEDP↑ LVEF-	Being good for PAH secondary to HFpEF studies	
Mouse	2017	Andreas B Gevaert	WD; 1%NaCl	8 weeks	24 weeks	SAM	NA	Female	EDPVR↑ LVEDP↑ Tau↑ E/E'↑ BNP↑	Being useful for studying age-related HFpEF	
Mouse	2017	Xiaochen He	NA	NA	48 weeks	NA	Sirt3 ECKO	Female	IVRT↑ MPI↑ E’↓ E/E’↑		
Mouse	2018	Guodong *Pan*	60%HFD	12 weeks	18 weeks	C57BL/6	ALDH2×2	Male	EF- CRR↓ RD↓ BNP↑		
Mouse	2018	Yuan Liu	45%HFD	3 weeks	18 weeks	C57BL/6	NA	Male	LVEDP↑ -dp/dtmin↓ Tau↑ EDPVR↑		
Mouse	2018	Linda Alex	NA	8 weeks	6 months	C57BL/6J	db/db	Male/Female	LVEF- E/E’↑ E/A- LVEDP↑		
Mouse	2019	Gabriele G Schiattarella	60%HFD; l-NAME (0.5 g/L) × 15 weeks	8–12 weeks	15 weeks	C57BL/6N	NA	Male	LVEF- GLS%↓ E/E’↑ LW (wet/dry)↑ HW/TL↑ RD↓	Short model-establishing period and prolonged EF preservation, being good for exploring the pathophysiological mechanisms of HFpEF.	
Mouse	2019	Keyvan Yousefi	Col4a3−/−	NA	2 months	129J	Col4a3−/−	Male	IVRT↑ E/E’↑ E/A↓ MPI↑ GLS↓GCS↓ LW↑ LW (wet/dry)↑		
Mouse	2020	Masataka Kamiya	Ang II (1.4 mg/kg/day) × 4 weeks	8–9 weeks	4 weeks	C57BL/6J	NA	Male	LVEDP↑ EDPVR↑ LW/BW↑		
Mouse	2020	Coenraad Withaar	60%HFD; AngII × 4 weeks	72–88 weeks	12 weeks	C57BL/6J	NA	Female	GLS%↓ RPLSR↓	The gender difference is consistent with the clinical feature	
Mouse	2020	Hong-Jie Yang	Uninephrectom; d-aldosterone (0.15 mg/h) × 4 weeks	8–10 weeks	4 weeks	C57BL/6	NA	Male	HW/BW↑ LW/BW↑-dp/dtmin↓ Tau↑ EDPVR-k↑	Shortening of modeling period	Being difficult to operate
Mouse	2020	Jason D Roh	NA	24–26 months	NA	C57BL/6	NA	Male	LVEDV↑ Tau↑ HW/TL↑ LW/BW↑		
Mouse	2021	Liyun Zhang	Uninephrectom; d-aldosterone (0.15 mg/h) × 4 weeks	8–10 weeks	4 weeks	C57BL/6	NA	Male	LVEF- HW/BW↑ -dp/dtmin↓ EDPVR-k↑ Tau↑		
Mouse	2021	Yan Deng	HFD; DOCP (75 mg/kg) × 4 weeks	12 weeks	13 months	C57BL/6J	NA	Male	BNP↑ -dp/dtmin↓ EDV↓ EDPVR↑ ERT↓	Providing clues to targeting mitochondria-inflammation circuit to mitigate HFpEF.	
Mouse	2021	Yuto Monma	NA	12 weeks	20 weeks	C57BL/6J	db/db	Male	LAD↑LVEF- LVEDP↑ -dp/dtmin↓ EDPVR-k↑		
Rat	2018	Felix Hohendanner	NA	21 weeks	NA	ZSF1-obese	NA	NA	LVEF-LVEDP↑ LAEF↑ LA↑		
Rat	2019	Alec Davila	NA	8 weeks	12 weeks	ZSF1-obese	NA	Male	LVEF- E/A↓DT↑		
Rat	2019	Fangbo Bing	8%NaCl	7 weeks	7 weeks	DSS	NA	Male	LVEDP↑E/A↓LLSR↓ SCSR↓		
Rat	2020	Zhongjie Yin	8%NaCl	7 weeks	7 weeks	DSS	NA	Male	LVEDP↑ ED Cauchy stress↑		
Rat	2020	Wenxi Zhang	8%NaCl	7 weeks		DSS	NA	Male	E/A↓ E/E’↑ HW/BW↑ LW/BW↑		
Rat	2020	Justine Dhot	Overexpression of endothelial β3-AR	0	45 weeks	SD	Tgβ3	Male	E/A↑ LA↑ LVEDP↑		
Rat	2020	Thassio Ricardo Ribeiro Mesquita	NA	0	21–24 months	Fischer F344	NA	Female	E/A↓ E/E’↑ LW/BW↑ LAD↑	Being helpful in exploring the relationship between AF and HFpEF	Self-terminating, unlike human disease
Rat	2020	Antje Schauer	NA	5 weeks	27 weeks	ZSF1-obese	NA	NA	E/E’↑ LVEF- LVEDP↑ BNP↑		
Rat	2020	Sin-Hee Park	NA	12 weeks	6 weeks	ZSF1-obese	NA	NA	PWT↑ LVEF-		
Rat	2021	Antje Schauer	NA	20 weeks	NA	ZSF1-obese	NA	Female	NT-proBNP↑ E/E’↑ LVEDP↑ LVPW↑ dP/dtmin↑		
Rat	2021	Taijyu Satoh	NA	8 weeks	12 weeks	ZSF1-obese	NA	NA	LVEDP↑ exercise tolerance↓ LVPWd↑ E/A↓		
Dog	2013	Nazha Hamdani	Bilateral renal wrapping	8–12 years	8 weeks	NA	NA	NA	MyD↑ LVEF-		Being expensive and difficult to obtain old animals
Pig	2017	Barry A Borlaug	HFD (2% cholesterol and 15% lard) × 6 weeks; unilateral renal artery coiling	NA	16 weeks	NA	NA	NA	LV mass↑ EF- EDV↓ Myocardial perfusion↓		
Pig	2018	Oana Sorop	DM (STZ-50 mg/kg/day × 3 days); HC(HFD) HT (renal artery embolization)	2–3 months	6 months	Yorkshire/Landrace	NA	Female	collagen↑ Fmax↑ Fpas↑ E/A↓ EDE↑ LVEDV↓ EF-		
Pig	2019	Nannan Zhang	Ang II (0.015 mg/h); DOCA (100 mg/kg) × 9 weeks; WD	39 weeks	18 weeks	Landrace	NA	Female	IVSd↑ LVPW↑ LA↑ LV + dp/dt↑ PASP↑ PADP↑ PCWP↑ E/E'↑ DT↑ IVRT↑ EDPVR↑ LV-dp/dt↑		
Pig	2016–2020	Ursula Reiter Christian Mühlfeld	DOCA (100 mg/kg) × 90 days; High-salt High-sugar High-potassium	NA	12 weeks	Landrace	NA	Female	IVRT↑ LAV↑ (E/E′)/EDV↑ S/D↓ MPR↓ torsion ratemax↑ V_V_(coll/lv)↑ V_V_(coll/int)↑ V (myo/lv)↑ V (col/lv)↑ V (ves/lv) ↑		

Abbreviations: E, mitral inflow E wave; A, mitral inflow A wave; E’, median mitral annular early diastolic velocity; DT, deceleration time; IVRT, isovolumetric relaxation time; HFD, high fat diet; DOCA, desoxycorticosterone; LVEDP, left ventricular end-diastolic pressure; -dp/dtmin, left ventricular minimum rates of pressure rise; Tau, left ventricular relaxation time constant; EDPVR, end diastolic pressure–volume relationship; k, constant of an exponential curve fit of the EDPVR; LVEDV, left ventricular end diastolic volume; HW/TL, heart weight/tibia length; HW/BW, heart weight/body weight; LW/BW, lung weight/body weight; GLS%, global longitudinal systolic strain; RPLSR, reverse peak longitudinal strain rate; l-NAME, Nω-Nitro-l-arginine methyl ester hydrochloride; NT-proBNP, N-terminal prohormone of brain natriuretic peptide; LVEF, left ventricular ejection fraction; ERT, endurance running time; RD, running distance; RVSP, right ventricular systolic pressure; mPAP, mean pulmonary arterial pressure; LAD, left atrial dimension; WD, western diet; Col4a3,collagen type IV, alpha 3 chain; ZSF1,Zucker diabetic fatty/Spontaneously hypertensive heart failure F1 hybrid; PWT, left ventricular posterior diastolic wall thickness; LVPWd, left ventricular posterior diastolic wall; SAM, Senescence-accelerated mice; STZ, streptozotocin; ED, cauchy stress, end diastolic Cauchy stress; LLSR, peak longitudinal strain rates; SCSR, peak circumferential strain rates; GCS, global circumferential strain; LAV, left atrial volume; S, maximal systolic pulmonary venous peak velocity; D, early diastolic pulmonary venous peak velocity; MPR, stress-to-rest myocardial perfusion reserve; torsion ratemax, maximal left ventricular torsion; VV(coll/lv),volume fraction of collagen with respect to the myocardium; VV(coll/int),volume fraction of collagen with respect to the interstitium; MyD, cardiomyocyte diameter; Fmax, maximal force; Fpas, passive force; EDE, End-diastolic elastance (slope of EDPVR, sEDPVR); PAH, pulmonary arterial hypertension; HFpEF, heart failure with preserved ejection; AF, atrial fibrilla.

## Small Animal Models of HFpEF

Mice are the most commonly used animal models in HF studies because they share most of their genes with humans, and approximately 85% of the protein-coding regions of the human genome can be found in the mouse genome. Mice also have the advantages of short reproductive cycle, low feeding cost, and being ready to gene editing. At present, some rodent HFpEF models have been constructed through chemical agents, diet, surgical intervention, genome editing and so on.

### How to Evaluate Cardiac Function of Small Animals

To build the animal models of HFpEF, tools to assess the atrial, ventricular or overall cardiac function of small animals should be established first. However, imaging or functional modalities are limited in small animals for estimating cardiac function. Up to date, aggressive cardiac pressure-volume loop analysis has been deemed as the “gold standard” in assessment of load-dependent and load-independent cardiac function. After the preparation of anesthesia and mechanical ventilation, conductance catheter had to be inserted through rodents’ apical stab or right carotid artery, which generally end up at the cost of termination of modeling. It was suggested that transient aortic occlusion can be used to alter afterload, whereas transient inferior Vena Cava occlusion can be used to alter preload. Hemodynamic parameters such as LV end-systolic pressure, LV end-diastolic pressure (LVEDP), peak positive LV dp/dt, peak negative LV dp/dt, time constant relaxation (tau), stroke volume, and cardiac output could be analyzed by pressure-volume conductance catheter technique (Warren, 1974; Pacher et al., 2008; Abraham and Mao, 2015).

Transthoracic echocardiography (TTE) specific to small animals (Fayssoil and Tournoux, 2013; Schnelle et al., 2018; Wang et al., 2018) were performed to evaluate ventricular function of small animals, with the advantage of non-aggressive modality. It is pivotal for evaluating the preservation of ventricular function and stability of HFpEF phenotype in time course studies. Myocardial systolic function in small animals assessed by echocardiography were ventricular EF, fraction shortening (FS) and +dp/dtmax. Impaired diastolic function in echocardiographic transmitral flow profile showed a “restrictive” filling pattern with increased early (E-wave) and decreased late (A-wave) diastolic transmitral filling velocities. Augmented Doppler E/E’ and left atrial diameter, rapid deceleration of the early filling wave, isovolumic relaxation time (IVRT) as well as diastolic reverse peak longitudinal strain rate (RPLSR) were important to estimate diastolic dysfunction.

In addition to hemodynamic and imaging measurements, exercise stress and energy metabolism can be used to evaluate cardiovascular function when taking mouse physiology into account. Therefore, the mouse-graded maximal exercise testing (GXTm) was designed by staged increases in inclination as speed progress until exercise exhaustion. During exercise test, metabolic chamber encloses the treadmill that mice run on. Run time, maximum run speed, oxygen consumption (VO2), carbon dioxide expiration (VCO2), and VCO2/VO2 could be generated from the gas exchange occurring inside the metabolic chamber. Therefore, GXTm could be used as a functional assessment to determine the cardio-metabolic phenotype of various small animal models (Petrosino et al., 2016).

### Pharmacological Agents or Diet-Induced HFpEF Models

Hypertension, the chronic pathological stress resulting in cardiac hypertrophy and fibrosis, as well as renal dysfunction, is an important metabolic risk factor for HFpEF incidence. Angiotensin II (Ang II), a vasoactive peptide, is known to regulate physiological blood pressure and is one of main players in hypertension development (Forrester et al., 2018). Ang II is applied for studying hypertension-related heart diseases (Peng et al., 2011; Valero-Munoz et al., 2017; Brenes-Castro et al., 2018). Kamiya et al. (2021) found that C57BL/6J mice treated with Ang II showed a significant increase in LVEDP and end-diastolic pressure-volume at the 4th week. Significant fibrosis and cardiac hypertrophy were also observed in the Ang II group, with no significant difference in LVEF and E/A. They also found that Ang II-mediated cardiac fibrosis was dependent on a cardiac cytokine, transforming growth factor (TGF)-β1. However, ANG II has also been used to establish animal models for HFrEF (Pan G et al., 2018). Therefore, whether ANG II a pharmacological stimulus for HFpEF modeling or eventually develops into a HFrEF model largely depends on modeling time.

Obesity induces metabolic and inflammatory disturbances and is linked to incidence of HFpEF (Tabucanon et al., 2020; Mishra and Kass, 2021). After 18 weeks of high-fat diet (HFD) on 45% fat only, C57/B6 mice showed impaired diastolic function and increased ratio of wet lung weight/dry lung weight, suggesting pulmonary congestion. And high-fat-fed mice showed hyperglycaemia (Liu et al., 2018). Echocardiography implied that there was no difference in systolic function between normal diet group (ND) and HFD group, and values of LVEF in both groups were normal. LVEDP and Tau index were increased in HFD group, whereas -dp/dt was decreased. Mechanistically, HFD induced myocardial insults such as oxidative stress and apoptosis. Melatonin (N-acetyl-5-methoxytryptamine), the neurohormone that maintains circadian rhythms, is involved in the regulation of blood pressure (Stauch et al., 2019). It was found that melatonin protected the damaged myocardium and improved HFpEF phenotype by promoting fat-derived CTRP3 secretion. This model sheds insight on obesity-related HFpEF and indicates the potential role of melatonin in improving cardiac diastolic dysfunction in HFpEF.

### Two-Hit or Multi-Hit HFpEF Models

HFpEF is a multi-etiological and complex clinical syndrome in clinical reality. Many prediction models for HFpEF incidence and prognosis were proposed recently, with a spectrum of clinical predictors such as obesity, old age, anemia, elevated blood pressure, renal dysfunction, atrial fibrillation and pulmonary hypertension (Reddy et al., 2018; Zhang J. X et al., 2019; Liang et al., 2021). As to an individual patient, the interaction of different pathological processes might contribute to HFpEF development. Therefore, two-hit or multi-hit models using the combined risks of metabolic syndrome and aging were introduced to establish small animal models of HFpEF. HFD with 60% fat and Nω-Nitro-l-arginine methyl ester hydrochloride (l-NAME) increased blood pressure and reduced glucose tolerance in C57BL/6N mice, leading to HFpEF phenotype such as poor left ventricular overall longitudinal strain % (GLS%), pulmonary edema, myocardial hypertrophy, cardiac fibrosis, increased aortic stiffness, decreased exercise endurance and the mitral flow deceleration time (Schiattarella et al., 2019). Notably, murine LVEF retention was observed in this model during the prolonged time course at the 5th, 15th, 24th, 50th week. Thus, considering a relatively short model-establishing period and prolonged EF preservation, this model is relatively ideal for studying pathophysiological mechanisms underlying HFpEF.

Given that aging is a pathological factor in the development of HFpEF, female, aged (18–22 months) C57BL/6J mice were administered with 60% HFD for 12 weeks and Ang II for the last 4 weeks (Withaar et al., 2020). Metabolic disorder related HFpEF phenotypes such as obesity, impaired glucose tolerance, and inflammation were observed in aging mice by HFD + Ang II intervention. HFD + Ang II mice demonstrated cardiac concentric hypertrophy assessed by echocardiography, with significantly decreased GLS% and diastolic RPLSR, suggesting that the diastolic ability of this group of mice was impaired. RNA-sequencing showed that in HFD + ANG II group, the expression of TGF families was upregulated. Intriguingly, the researchers found that male mice treated with HFD + Ang II showed HFrEF phenotype whereas female mice tended to be HFpEF phenotype. The gender difference is consistent with the clinical feature that women are more likely to develop HFpEF.

In addition to Ang II and l-NAME, deoxycorticosterone acetate (DOCA) and desoxycorticosterone pivalate (DOCP) were used as agent for increasing blood pressure and constructing two-hit or multi-hit model of hypertension. An obese, senile, and hypertensive murine model induced by HFD for 13 months combined DOCP in the last month successfully produced HFpEF phenotypes (Deng et al., 2021). Mice in HFD + DOCP group showed impaired glucose tolerance, myocardial hypertrophy, exercise intolerance, cardiac fibrosis, pulmonary edema, decreased -/dt, and significantly decreased end-diastolic volume. The HFpEF mice exhibited overproduction of IL-1β/IL-18. Mechanistically, the interaction between mitochondrial hyperacetylation and inflammation was a key driver in the pathogenesis of obesity and hypertension-related HFpEF, which can be salvaged by elevating the abundance of β-OHB *in vivo* and *in vitro*. These findings provide clues to targeting mitochondria-inflammation circuit to mitigate HFpEF.

### Surgery-Induced HFpEF Models

Patients with chronic kidney diseases (CKD) and renal dysfunction represent a subpopulation of HFpEF with worse outcome (Kirkman et al., 2021). To study CKD-related HFpEF, uninephrectomy of mice is gradually adopted as a surgery to induce HFpEF phenotype (Valero-Munoz et al., 2016; Yang et al., 2020). 4 weeks after uninephrectomy, aldosterone or DOCA were administered, C57BL/6J mice manifested with pulmonary edema, increased mitral valve velocity E and A, and significantly reduced IVRT and deceleration time (DT). Mice that underwent uninephrectomy and received a continuous infusion of d-aldosterone (0.15 mg/h) for 4 weeks displayed HFpEF phenotype assessed by ultrasound and Millar catheter transducer (Zhang et al., 2021). HFpEF mice showed markedly upregulated levels of plasma IL-1β, IL-6, and tumor necrosis factor (TNF)-α. The advantage of surgical intervention is the shortening of modeling period. However, the surgical procedure is a challenge to the operators. Other limitations are bias between groups and poor reproducibility of different research groups.

### Gene Editing Induced HFpEF Models

Some gene-edited mice were predisposed to develop HFpEF due to altered genotypes. Leptin resistant db/db mice constructed under the C57BL/6J background spontaneously developed severe obesity and diabetes (Chen et al., 1996; Chen et al., 2012; Alex et al., 2018; Monma et al., 2020). Alex et al. (2018) found that at 6 months of age, both male and female db/db mice developed HFpEF, showed as cardiac hypertrophy, cardiac fibrosis, and significantly higher E/E’ than WT group. In addition, the influence of sex difference on diastolic function in db/db mice was also studied. LVEDP of female db/db mice was significantly higher than that of female WT group. Shimokawa and his colleagues reported that cardiac diastolic function of db/db mice were impaired at 20 weeks, assessed with transthoracic echocardiography and invasive haemodynamic analysis. Interestingly, low-intensity pulsed ultrasound (LIPUS) therapy ameliorates cardiac diastolic dysfunction through improvement of eNOS-NO-cGMP-PKG pathway and cardiomyocyte Ca2+-handling system. They indicated that the LIPUS therapy significantly upregulated the expression of SERCA2a and phosphorylated eNOS (Monma et al., 2021). Some studies have reported significant reductions in LVEF in db/db mice compared with control (Barouch et al., 2006; Shen et al., 2009; Plante et al., 2015). Others have not shown any functional or structural differences between db/db mice and age-matched control (Ko et al., 2017). Due to these conflicting conclusions, further research is needed to determine whether db/db mice are really suitable for HFpEF study.

Other gene-edited mice with phenotypes of HFpEF have been reported. Sirtuin 3 plays a critical role in the regulation of endothelial glycolysis and angiogenesis. Research from Chen’s group showed loss of Sirtuin 3 in endothelial cells impaired myocardial microcirculation and induced diastolic dysfunction (He et al., 2017). Col4a3^−/−^ mice was used in a model of human Alport syndrome with hypertension and kidney failure phenotype. Col4a3^−/−^ mice manifested diastolic dysfunction, cardiac hypertrophy, fibrosis and pulmonary edema via upregulation of osteopontin (OPN). 2-Oxo-Glutarate Dehydrogenase-like (OGDHL), a mitochondrial protein involved in metabolic substrate fluxes and signaling, mediates OPN-induced mitochondrial dysfunction in promoting a HFpEF-like cardiac phenotype in this model (Yousefi et al., 2019). Aldehyde dehydrogenase 2 (ALDH2) is a mitochondrial aldehyde detoxifying enzyme in the metabolism of acetaldehyde, especially lipid peroxidation-derived reactive aldehyde under oxidative stress. ALDH2 rs671 polymorphism caused reduced activities of ALDH2. We found patients with ALDH2 rs671 polymorphism were associated with higher risk of HFpEF (Zhang N et al., 2019; Xia et al., 2020). HFD-fed type 2 diabetic ALDH2*2 mutant mice (Pan X et al., 2018) or ALDH2 knockout mice with diabetes induced by streptozotocin (Wang et al., 2016) exhibited HFpEF phenotypes when measured by cardiac echo stress test. Altogether, the limitation of knockout mouse models were the long reproductive cycle and high feeding cost.

### Strain Induced HFpEF Models

The mouse strain had an important influence on the construction of HFpEF model. Meng et al. (2017) reported that AKR/J mice fed a 60% fat energy diet for 20 weeks showed ventricular hypertrophy, pulmonary artery remodeling, glucose intolerance, and increased LVEDP. And HFD-treated AKR/J mice had higher body weights and glycated hemoglobin levels. This model may be useful in study of pulmonary arterial hypertension secondary to HFpEF. Gevaert et al. found that another AKR/J background accelerated aging mice (SAM) showed HFpEF phenotype after HFD. The cardiac alteration of SAM + HFD at 24 weeks of age was diastolic dysfunction, left ventricular hypertrophy, left atrial dilatation, and interstitial fibrosis. Exercise capacity was reduced and lung weight increased in SAM + HFD mice (Gevaert et al., 2017). This model is useful for studying age-related HFpEF. However, compared with the physiologically senile mice with C57BL/6 background (Roh et al., 2020), the accelerated aging state of SAM-HFD model may affect the pathophysiological studies of HFpEF.

### Rat Models of HFpEF

Obese diabetic Zucker fatty/spontaneously hypertensive HF F1 hybrid (ZSF1-OB) rats exhibit spontaneous type 2 diabetes, diabetic kidney disease, hypertension, hyperlipidemia, obesity and metabolic syndrome features (Hohendanner et al., 2018; Davila et al., 2019; Leite et al., 2019; Park et al., 2020; Schauer et al., 2020). A number of studies demonstrated that ZSF1-OB had cardiac manifestations such as normal LVEF, increased E/A, left atrial enlargement, elevated LVEDP, upward EDPVR curve, and reduced activity tolerance (Leite et al., 2019; Satoh et al., 2021; Schauer et al., 2021). In addition, compared with lean ZSF1(ZSF1-LN) in the control group, ZSF1-OB displayed significant aortic remodeling and arterial collagen deposition, central vascular remodeling, which aggravated the hypertensive phenotype and promoted HFpEF. ZSF1-OB is a commonly used rat model of HFpEF. However, because ZSF1 rats were constructed by crossing leptin non-hypertensive Zucker diabetic obese female rats (ZDF, +/FA) and leptin spontaneous hypertensive male rats with HF (SHHF/MCC, +/FACP), the ZSF1 rats had no ability to reproduce, which limited the generalization of this model. Bing et al. found that salt-sensitive rats (DSS) on a high-salt diet (HS) for 7 weeks showed increased left ventricular diastolic stiffness and decreased left ventricular relaxation (Bing et al., 2020; Yin et al., 2020). Zhang et al. revealed that compared with the low-salt diet group (DSS + LS), the endocardial and epicardial radial strain peaks of DSS + HS rats were significantly reduced (Zhang et al., 2020), accompanied by increased lung and kidney mass and impaired diastolic function. Additionally, glucose transporter 1 expression and cardiac glycolysis were increased. And a marked increase in proton production was observed. It should be noted that this model only represents part of HFpEF secondary to hypertension and is suitable for the study of the mechanisms of HFpEF induced by hypertension. A transgenic rat model that overexpresses human β3 -adrenoceptor (hβ3 -AR) in the endothelium was reported to assume increasing diastolic dysfunction with age (Dhot et al., 2020). Mesquita found reduced E/A, increased E/E’, increased lung weight, cardiac hypertrophy, atrial enlargement, and decreased motor ability in female Fischer F344 rats aged 21–24 months (Mesquita et al., 2020). In addition to diastolic dysfunction, this rat model also has a propensity for atrial fibrillation, which is helpful in exploring the causal relationship between atrial fibrillation and HFpEF. However, ion channel expression in the model was not measured, and AF in this rodent model is self-terminating, unlike human disease.

## Large Animal Models of HFpEF

Although rodents are widely used as models to study the mechanisms of human heart diseases, they do not fully meet the needs of heart disease models due to limited information obtained by measurements of heart anatomic structures and function. The construction of large animal models using sheep, pigs or dogs provides approaches to solve this problem. But ethical concerns, cost, study duration and difficulties in gene editing limit large animal models of HFpEF.

### How to Evaluate Cardiac Function of Large Animals

Similar to the evaluation of cardiac function of humans, clinical modalities, such as ECG-gated multidetector computed tomography (MDCT), cardiovascular magnetic resonance (CMR), echocardiography and cardiac catheter were used to assess functional, metabolic, and structural changes of myocardium in large animal models (LeBlanc et al., 2017). Myocardial perfusion and myocardial work could be calculated by positron emission tomography (PET), in combination with [(15)O] water and oxygen consumption using [(11)C] acetate (Tarkia et al., 2015). CMR at rest, as well as during dobutamine stress, were applied in estimate left ventricular/atrial function and myocardial mass in large animals. Additionally, strains and torsion were evaluated from tagged cine imaging. By virtue of 4D phase-contrast measurements, blood flow and peak velocities, including transmitral early-diastolic (E) and myocardial tissue (E’) velocities, as well as coronary sinus blood flow were assessed in large animals, making it possible to estimate myocardial perfusion reserve from stress-to-rest time-averaged coronary sinus flow (Reiter et al., 2016). Although these non-invasive methods provide a variety of choices to assess cardiac pathophysiological alterations, measurement of hemodynamics by cardiac catheter is still the golden standard to assess LV compliance and relaxation in HFpEF.

### Surgery-Induced HFpEF Models

Hypertension-induced LV hypertrophy (OHT) was built by bilateral kidney ligation on elderly dogs that developed hypertension 8 weeks later. Compared with the control group, the OHT group showed HFpEF phenotype as increased left ventricular mass, myocardial hypertrophy, abnormal Tau index and BNP level. Mechanistically, functional impairment of sarcomeres may be an early event of HFpEF as this model suggested, mainly manifesting hypophosphorylation of myofilament proteins and increased Ca^2+^ sensitivity (Hamdani et al., 2013). However, the requirement for old animals (8–13 years old) makes this model relatively impractical and expensive.

Unilateral renal artery stenosis with renovascular hypertension (RVHT) was constructed by placing an irritant coil in the main renal artery under fluoroscopy, which leads to a gradual narrowing of the renal artery. In combination of HFD, this model displayed increased LV mass and diastolic dysfunction. In the HFD + RVHT pig model, the elevation in LV filling pressures with volume loading was mitigated by percutaneous approach using pericardial resection (Borlaug et al., 2017). Myocardial expression of TNF-α and IL-6 was upregulated in RVHT pigs. Furthermore, in pigs with RVHT, percutaneous transluminal renal angioplasty, along with intra-renal. Bendavia, a mitochondrial targeted peptide, can improve cardiac function in RVHT pigs. Mesenchymal stem cells delivery restored renal and cardiac function (Eirin et al., 2014; Eirin et al., 2015). This RVHT model hints the possibility of therapeutic options for patients with this specific subtype of HFpEF.

### Multi-Hit HFpEF Models

Thirty-nine-week-old female landrace pigs that were delivered with Ang II and DOCA, and fed with HFD exhibited a significant increase in the LV thickness compared with the Normal group. Total cholesterol (TC), triglyceride (TG) and low-density lipoprotein (LDL) were markedly increased in HFpEF group. As expected, the levels of IL-6 and TNF-α were elevated in the aortas of HFpEF pigs. And the plasma BNP, plasma epinephrine, norepinephrine and Ang II concentrations in HFpEF pigs were strongly increased when compared with the normal group. Invasive right cardiac catheterization was performed to show that PASP and PCWP were significantly higher in HFpEF pigs compared with the normal group, especially at the 18th week (Zhang J. X et al., 2019).

HFpEF was induced in pigs by subcutaneous implantation of DOCA pellets along with a high-salt, high-sugar, high-potassium diet over 3 months. When compared with weight-matched normal pigs, cardiomyocyte changes were similar in subepicardial, midmyocardial and subendocardial regions but DOCA-induced changes in the interstitium appeared to be more pronounced in the subendocardial ventricular wall layers (Muhlfeld et al., 2020). The results of this study suggest a pivotal role of the subendocardial interstitium in the pathogenesis of HFpEF. Three comorbidities, including DM (induced by streptozotocin), hypercholesterolemia (produced by HFD), and hypertension (resulted from renal artery embolization), were established in female swine. The combination treatment leads to systemic inflammation, myocardial oxidative stress, and coronary microvascular dysfunction, which associate with myocardial stiffening and LV diastolic dysfunction with preserved EF (Sorop et al., 2018).

## Summary and Perspectives

**FIGURE 2:**
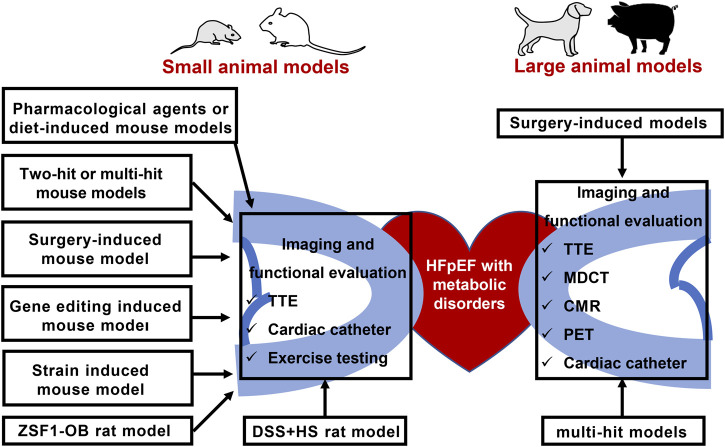
Animal models of HFpEF and metabolic disorders, as well as the imaging and functional modalities for animal cardiac assessment. TTE: Transthoracic echocardiography; HFpEF: heart failure with preserved ejection fraction; ZSF1-OB: Obese diabetic Zucker fatty/spontaneously hypertensive heart failure F1 hybrid rats; DSS + HS: salt-sensitive rats on a high-salt diet; PET: positron emission tomography; MDCT: multidetector computed tomography; CMR: cardiovascular magnetic resonance.

In this review, we summarized the published animal models of HFpEF and metabolic disorders, as well as the imaging and functional modalities for animal cardiac assessment (Figure 2). First and foremost, the reliability and stability of the existing HFpEF animal model should be considered, as well as the optimized evaluation approaches of HFpEF. Next, diagnostic criteria of HFpEF patients is used as a reference to evaluate success of animal phenotype. Therefore, researchers should pay attention to the physiological difference of the animals and human beings. Lastly, targeting cardiac metabolism has great potentials to ameliorate HFpEF prognosis. EMPEROR-Preserved study released that sodium-glucose cotransporter 2 inhibitors empagliflozin reduced the combined risk of cardiovascular death or hospitalization for patients with HFpEF. It is noteworthy that some HFmrEF patients have been clinically diagnosed with HFpEF and the pathogenesis. Nonetheless, therapeutic effects of HFmrEF are similar to HFrEF indeed. 33% of the patients in EMPEROR-Preserved study had a baseline EF of 41–50% and were actually HFmrEF patients (Anker et al., 2020; Anker et al., 2021). Whether empagliflozin treatment benefits HFpEF patients with LVEF ≥50% is unknown. New trials enrolled patients with LVEF ≥50% are crucial to ascertain the efficacy of novel HFpEF therapies. Potential metabolic targets to attenuate animal myocardial fibrosis and improve cardiac function are needed for clinical translation in the future.
